# Upregulation of GRIM-19 inhibits the growth and invasion of human breast cancer cells

**DOI:** 10.3892/mmr.2021.12225

**Published:** 2021-06-16

**Authors:** Wei Zhang, Ye Du, Tong Jiang, Wei Geng, Jiuli Yuan, Duo Zhang

Mol Med Rep 12: 2919-2925, 2015; DOI: 10.3892/mmr.2015.3757

Following the publication of this paper, it was drawn to the Editors’ attention by a concerned reader that the western blotting data featured in Figs. 4B and 6B (wherein there was also a duplicated set of data bands) were strikingly similar to data appearing in different form in other articles at different research institutes. Owing to the fact that the contentious data in the above article were already under consideration for publication, or had already been published, elsewhere prior to its submission to Molecular Medicine Reports, the Editor has decided that this paper should be retracted from the Journal. The authors did not reply to indicate whether or not they agreed with the retraction of the paper. The Editor apologizes to the readership for any inconvenience caused.

